# Resveratrol enhances the clearance of mitochondrial damage by vitrification and improves the development of vitrified-warmed bovine embryos

**DOI:** 10.1371/journal.pone.0204571

**Published:** 2018-10-18

**Authors:** Tomotaka Hara, Airi Kin, Sogo Aoki, Shinsuke Nakamura, Koumei Shirasuna, Takehito Kuwayama, Hisataka Iwata

**Affiliations:** Department of Animal Science, Tokyo University of Agriculture, Atsugi, Kanagawa, Japan; University of Florida, UNITED STATES

## Abstract

The present study investigated the vitrification-induced deterioration of mitochondrial functions that may reduce the developmental ability of post-warming bovine embryos. In addition, the effect of supplementation of the culture medium with resveratrol on the mitochondrial functions and post-warming embryonic development was examined. Two days after in vitro fertilization, embryos with 8–12 cells (referred to hereafter as 8-cell embryos) were vitrified and warmed, followed by in vitro incubation for 5 days in a culture medium containing either the vehicle or 0.5 μM resveratrol. Vitrification reduced embryonic development until the blastocyst stage, reduced the ATP content of embryos, and impaired the mitochondrial genome integrity, as determined by real-time polymerase chain reaction. Although the total cell number and mitochondrial DNA copy number (Mt-number) of blastocysts were low in the vitrified embryos, the Mt-number per blastomere was similar among the blastocysts derived from fresh (non-vitrified) and vitrified-warmed embryos. Supplementation of the culture medium with resveratrol enhanced the post-warming embryonic development and reduced the Mt-number and reactive oxygen species level in blastocysts and blastomeres without affecting the ATP content. An increase in the content of cell-free mitochondrial DNA in the spent culture medium was observed following cultivation of embryos with resveratrol. These results suggested that vitrification induces mitochondrial damages and that resveratrol may enhance the development of post-warming embryos and activates the degeneration of damaged mitochondria, as indicated by the increase in the cell-free mitochondrial DNA content in the spent culture medium and the decrease in the Mt-number of blastocysts and blastomeres.

## Introduction

Resveratrol (trans-3,5,4′-trihydroxystilbene), is a phytoalexin contained in many plant species such as grapes, peanuts, and berries, has received special attention worldwide for its potential use in cancer therapy and anti-aging strategies. Supplementation of culture media with resveratrol has been reported to enhance SIRT1 expression in oocytes and granulosa cells, thereby improving oocyte growth, maturation, and subsequent development [1–3]. SIRT1, a class III histone deacetylase, plays key roles in a variety of cellular processes, one of the most prominent one being the regulation of mitochondrial homeostasis [4]. In somatic cells, mitochondrial functions and quantity are strictly regulated by a quality control system [5–7], which is closely associated with mitochondrial homeostasis. For instance, carbonylcyanide m-chlorophenylhydrazone (CCCP)-induced mitochondrial dysfunction has been shown to activate mitochondrial degradation [8]. Moreover, CCCP treatment of porcine oocytes has been reported to induce mitochondrial dysfunction but subsequently increase mitochondrial biogenesis and degradation [9]. Treatment of porcine oocytes with resveratrol increased mitochondrial biogenesis and degradation in the oocytes, through SIRT1 activation and improved their developmental ability [10]. Moreover, CCCP-induced mitochondrial dysfunction has been found to differentially influence oocyte developmental ability and mitochondrial synthesis between young and aged cows; the differential mitochondrial response may be attributed to the differential activation of SIRT1 between the two age groups following CCCP treatment [11]. Based on these studies, it may be suggested that the up-regulation of SIRT1 expression induced by resveratrol may attenuate mitochondrial dysfunction in oocytes and embryos.

Cryopreservation of embryos and oocytes is an important procedure in assisted reproductive technology for humans and domestic animals. Cryopreservation enables the widespread utilization of genetically valuable animal embryos and increases the opportunities of pregnancy. However, a major challenge in the current cryopreservation technology is the compromised quality of oocytes and embryos after warming. For instance, the use of vitrified blastocyst-staged ovine embryos, instead of fresh ones, has led to low pregnancy rates (5.0–54.3%) [12]. Moreover, vitrified mouse oocytes have low developmental abiliies and mitochondrial dysfunction, owing to the abnormal mitochondrial localizations [13, 14]. Vitrification of porcine oocytes was shown to reduce the mitochondrial membrane potential and ATP content and decreased the developmental ability of the oocytes [15]. Furthermore, electron microscopy observation revealed the mitochondrial membrane damage and low electron density of the mitochondrial matrix in vitrified-warmed oocytes [15, 16]. Vitrification-induced mitochondrial damage and low survival rates were also reported for bovine blastocysts [17]. Therefore, the cryopreservation-induced mitochondrial dysfunctions may cause cryopreservation-associated deterioration of oocytes and embryos. Nevertheless, the fate of the damaged mitochondria in warmed oocytes and embryos remains unclear. As vitrified-warmed embryos and oocytes retain the developmental ability, the damaged mitochondria may recover from cryo-injury or get eliminated from the cells during the post-warming developmental period. Although the monitoring of mitochondrial degeneration in embryos is difficult, a previous study showed living granulosa cells to actively secrete mitochondrial DNA into the spent culture medium in response to the induction of mitochondrial dysfunctions [18]. Furthermore, cell-free DNA was detected in the spent culture medium of fragmented embryos [19], suggesting the usefulness of the mitochondrial DNA content in the spent culture medium as a marker of mitochondrial dynamics in embryos. In the present study, we investigated if the incubation of vitrified-warmed embryos in a resveratrol-containing culture medium may support the recovery of embryos from cryoinjury and enhanced their developmental ability through the degeneration of damaged mitochondria.

## Materials and methods

### Chemicals and culture media

All the chemicals were purchased from Nacalai Tesque (Kyoto, Japan), unless otherwise indicated. Medium 199 (Gibco, Grand Island, NY, USA) supplemented with 10% fetal calf serum (FCS; 5703H; ICN Pharmaceuticals, Costa, Mesa, CA) and 5 mM taurine was used for the in vitro maturation (IVM) of oocytes. In vitro fertilization (IVF) and in vitro culture (IVC) media were prepared using synthetic oviductal fluid (SOF) [20] with minor modifications, as previously reported [21]. The IVF medium comprised SOF containing 5 mg/mL of fatty acid-free bovine serum albumin (BSA) and 10 IU/mL heparin (Sigma-Aldrich, St. Louis, MO). IVC medium, which was used for the culture of oocytes during the initial 48 h of incubation post-fertilization comprised SOF with amino acids (Sigma-Aldrich, St, Louis, MO, USA), 1.5 mM glucose, and 1% FCS. The concentration of FCS in the culture medium increased from 1% to 5% during the incubation of oocytes from 48 h to 7 days after fertilization.

### Collection of ovaries and oocytes

Bovine ovaries were collected from a local slaughterhouse, stored at approximately 25 °C in phosphate-buffered saline (PBS) containing 10 mM glucose, 10 mM sucrose, and antibiotics, and were transported to the laboratory within 4 h. This study was approved by the Ethical Committee for Animal Experiment of Tokyo University of Agriculture. Cumulus-oocyte complexes (COCs) were collected from the ovaries using an 18-gauge needle connected to a syringe. The COCs were cultured in 100 μL of IVM medium (10 oocytes/drop) under paraffin oil (tissue culture grade) for 21 h at 38.5 °C in an atmosphere of 5% CO_2_ with maximum humidity.

### IVF and IVC of embryos

Frozen-thawed semen from a Japanese black bull (Livestock improvement Association of Japan, Tokyo, Japan) was washed with a discontinuous 30% to 60% Percoll gradient solution (GE Healthcare, Uppsala, Sweden) by centrifugation (800 ×g) for 10 min. After centrifugation, sperm cells (2 × 10^6^ cells/mL) were co-incubated with oocytes for 5 h in 100 μL of IVF medium (10–15 oocytes/drop). After fertilization, COCs were cultured in 100 μL of IVC medium (10–15 oocytes/drop). The fertilization and cultivation (2 days after fertilization) of COCs were performed at 38.5 °C in an atmosphere of 5% CO_2_ with maximum humidity. Cumulus cells surrounding the embryos were denuded to obtain embryos with 8–12 cells (referred to hereafter as 8-cell embryos) that were subsequently subjected to vitrification. In all the experiments, 10 of 8-cell embryos were individually cultured in a 5-μL droplet of IVC medium, and blastulation was examined under a stereo microscope on day 5 post-warming. Resveratrol was diluted in ethanol (vehicle) and added to the medium (2,000×) at a concentration of 0.5 μM. This concentration of resveratrol was selected based on the previous studies using bovine early embryos and blastocysts [22, 23]. Embryos cultured in the medium containing ethanol without resveratrol were used as the control groups.

### Vitrification and warming of embryos

As previously mentioned, 8-cell embryos were vitrified by an established procedure with minor modifications [24]. The 8-cell embryos (n = 5) were immersed in a base medium (HEPES-buffered TCM-199 containing 20% FCS) supplemented with 7.5% ethylene glycol (EG) and 7.5% dimethyl sulfoxide (DMSO; Wako Pure Chemicals, Osaka, Japan) for 10 min at room temperature for equilibration. The embryos were then immersed in a vitrification medium (base medium containing 15% EG, 15% DMSO, and 0.5 M sucrose) for 60 s. These embryos were loaded on to a Cryotop device (Kitazato Supply Co., Fujinomiya, Japan) and plunged into liquid nitrogen. After preservation for more than a day, embryos from the Cryotop device were gradually thawed in the base medium containing 1 M sucrose for 60 s at 38 °C, followed by a step-wise transfer into the base medium containing 0.5, 0.25, and 0.0 M sucrose for 3, 5, and 5 min, respectively.

### ATP assay

The ATP content of each embryos was measured based on the luminescence generated in an ATP-dependent luciferin-luciferase reaction (ATP Assay Kit; TOYO B-Net., Ltd., Tokyo, Japan) [25]. Luminescence was measured immediately after the reaction using a luminometer (Spark 10M; Tecan Japan Co., Ltd., Kanagawa, Japan). Embryos were transferred into 50 μL of distilled water and stored at −20 °C.

### Quantification of the Mt-number in blastocysts and blastomeres

Prior to DNA extraction, the total cell number in the blastocysts was determined under a fluorescence microscope (IX71; Olympus, Tokyo, Japan) by Hoechst-33342 staining (1.0 μg/mL). DNA from blastocysts was extracted by incubation in a lysis buffer (6 μL; 20 mM Tris, 0.4 mg/mL proteinase K, 0.9% Nonidet P-40, and 0.9% Tween-20) at 55 °C for 30 min, followed by incubation at 98 °C for 5 min. The Mt-number was determined by real-time polymerase chain reaction (PCR) using a Corbett Rotor Gene 6000 real-time rotary analyzer (Corbett Research, Sydney, Australia). Real-time PCR was performed at an initiation at 95 °C for 3 min, followed by 40 cycles of 98 °C for 5 s, and 59 °C for 11 s. The primer set was designed using Primer-BLAST (NC_006853.1: forward, 5’-GTAACCGCACACGCATTTGT-3’ and reverse, 5’-GGAATGAGGGAGGGAGGAGT-3’; for short sequence: 5858–6014, 157 bp). A standard curve was generated for each assay using 10-fold serial dilutions, representing copies of the external standard, which was the PCR product of the corresponding gene cloned into a vector using the Zero Blunt TOPO PCR Cloning Kit (Invitrogen). Prior to its use, this product was sequenced for confirmation. The Mt-number was divided by the cell number to determine the number of mitochondria per blastomere.

### Mitochondrial DNA integrity assay

Fresh non-vitrified and vitrified-warmed (n = 5 each) 8-cell stage embryos were transferred to 24 μL of DNA extraction buffer. Embryonic DNA was extracted using the protocol as described in the previous section. PCR was conducted by targeting two mitochondrial DNA sequences (long sequence: 736–7,156, 6,321 bp; forward primer: 5’-ATACCAAATAGGGTTAAATTCTAASTAA-3’, reverse primer: 5’-ATGATGAAAACTATTAGTATAACTGCTG-3’ and short sequence: 5,858–6,014, 157 bp; primers were similar to those used for mitochondrial DNA quantification). Each of the extracted DNA sample (6 μL) was used for real-time PCR. The average CT values from duplicate measurements were determined and used for the prediction of the Mt-number. The average Mt-number of fresh (non-vitrified) embryos was taken as 1.0, and the relative Mt-number of vitrified embryos was determined using the formula (2^fresh-CT^/2^vitified-CT^). PCR targeting the long mitochondrial sequence was performed at an initiation temperature of 95 °C for 10 min, followed by 40 cycles of 95 °C for 20 s, and 59 °C for 30 s, and 72 °C for 3 min. The PCR program, targeting the short mitochondrial sequence was the same as that was used for estimation of Mt-number of embryos.

### Cell-free DNA extraction from spent culture medium

On day 5 of IVC (day 7 post-IVF), blastocyst development was examined under a stereomicroscope, and the spent blastocyst culture medium was collected for DNA extraction. Culture medium samples were mixed with 5 μL of DNA extraction buffer (2×, 40 mM Tris, 0.8 mg/mL proteinase K, 1.8% Nonidet P-40, and 1.8% Tween-20) and incubated at 55 °C for 30 min, followed by an additional incubation step at 98 °C for 5 min. DNAs obtained from these medium samples were diluted up to six times and subsequently subjected to real-time PCR (protocol was similar to that described in the previous section) to assess the mitochondrial DNA content in the spent culture medium.

### Quantification of reactive oxygen species (ROS) level in blastocysts

The level of ROS in blastocysts were determined using CellROX Green Reagent (Thermo Fisher Scientific, Waltham, MA), according to the manufacturer’s instructions. Blastocysts were simultaneously stained with Hoechst-33342 and CellROX Green Reagent to determine the total cell number and ROS content, respectively. The fluorescence intensity of CellROX reagent was measured by Leica Application Suite Advanced Fluorescence (LAS AF) with a Leica DMI 6000B microscope (Leica Microsystems, Wetzlar, Germany) and quantified using ImageJ (NIH, Bethesda, MD). We calculated the ROS content per blastomere by dividing the intensity of CellROX reagent by the total cell number of blastocysts.

### Immunostaining

Vitrified-warmed embryos were cultured in IVC medium for 24 h. Embryos were then fixed in 4% paraformaldehyde for 1 day, and then immunostained, as previously described [10]. The primary antibodies used were rabbit anti-TOMM20 (1:200; Abcam, Cambridge, UK) and mouse anti-dsDNA (1:200; ab27156); secondary antibodies used were goat anti-rabbit IgG Alexa Fluor 555 (1:500; Cell Signaling Technology Inc., Danvers, MA) and goat anti-mouse IgG Alexa Fluor 488 (1:500; Cell Signaling). Embryos were mounted onto glass slides with an anti-fade reagent containing 4**′**,6-diamidino-2-phenylindole (DAPI). These embryos were observed under a Leica DMI 6000B microscope using LAS AF software (Leica, Wetzlar, Germany), and the fluorescence intensities were quantified using ImageJ software ((NIH, Bethesda, MD, USA). Twenty-seven embryos were immunostained for TOMM20 and the fluorescence intensity of each whole embryo was captured. Thirty-seven embryos were immunostained for double stranded DNA (dsDNA). The equatorial region of each embryo was captured in five slice (0.03 μm-thickness). Images were processed using 3D de-convolution software (Leica, Wetzlar, Germany). To quantitate the fluorescence intensity of cytoplasmic dsDNA, fluorescence intensity of the nucleus in each embryo was subtracted from that of the whole embryo. For each experiment, the nonspecific fluorescence intensity of negative control embryos stained without primary antibodies was also examined.

### Statistical analysis

Comparison of the data between the two groups was performed using Student’s t-test. The data among the three groups were analyzed using analysis of variance (ANOVA), followed by Tukey’s post-hoc test. Prior to the analysis, the developmental rate was subjected to arcsine transformation. A value of P < 0.05 was considered statistically significant.

## Results

### Vitrification reduced the ATP content and mitochondrial DNA integrity of post-warming embryos

**Fig 1:**
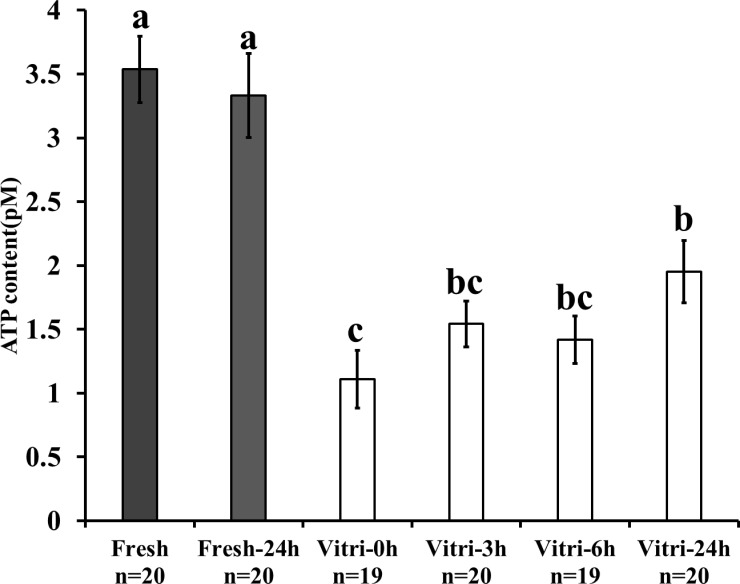
Effects of vitrification on the kinetics of ATP content in vitrified-warmed embryos. Fresh and vitrified-warmed embryos were incubated in IVC medium for 24 h. The ATP content of the post-warming embryos was measured at 0, 3, 6, and 24 h. Bars with different letters represent difference between treatments (P < 0.05). Data are presented as mean ± SEM.

**Fig 2:**
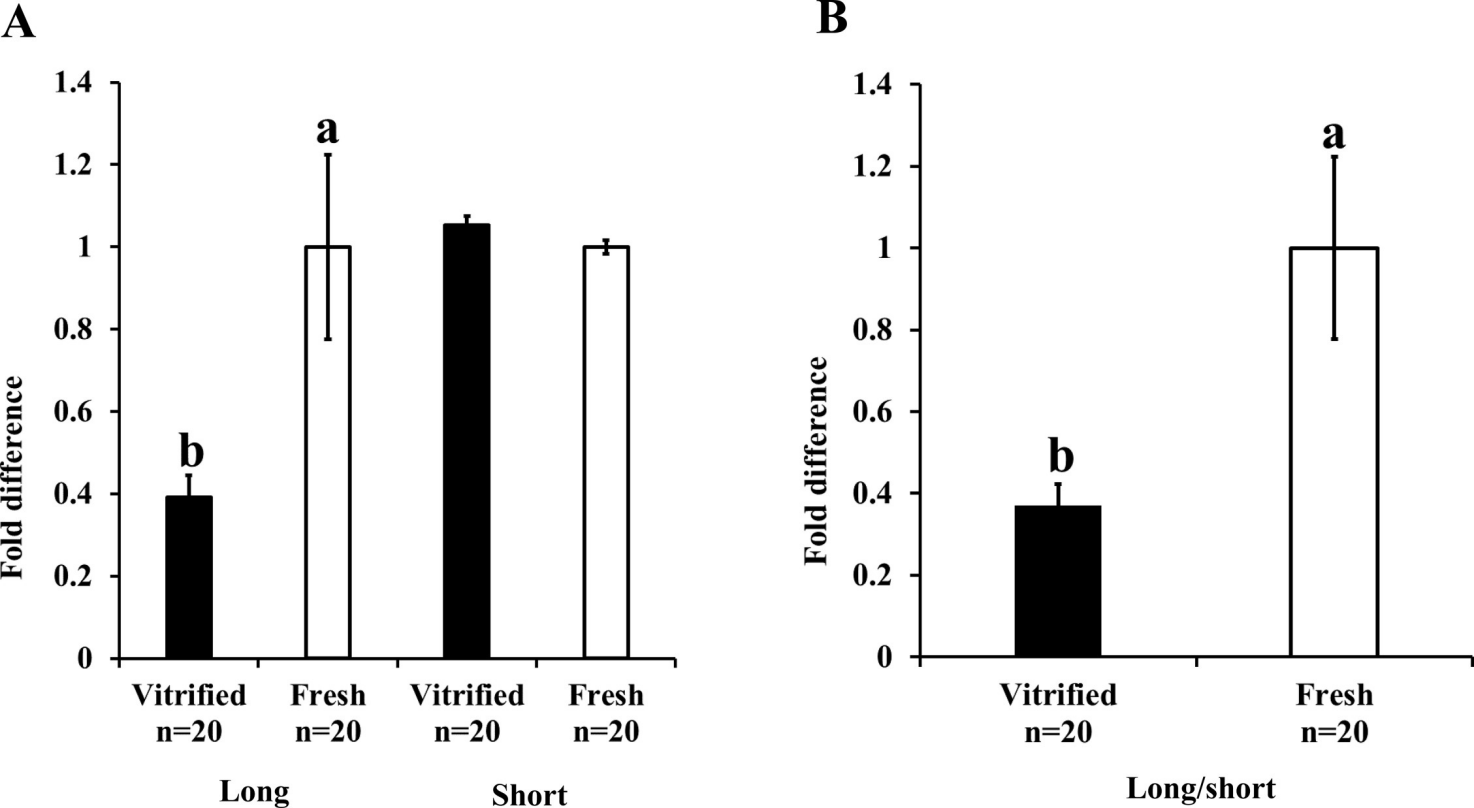
Effect of vitrification on the mitochondrial DNA integrity of embryos immediately after warming. The integrity of the mitochondrial genome was assessed according by the ratio of the Mt-number (long sequence/short sequence) predicted by real-time PCR targeting short and long mitochondrial sequences. A) Relative Mt-number predicted by PCR targeting the short and long mitochondrial sequences; the relative value for fresh embryos was taken as 1.0. B) Ratio of the Mt-numbers (Mt-number predicted by PCR targeting long sequence/Mt-number predicted by PCR targeting short sequence PCR). Value for fresh embryos was taken as 1.0. Bars with different letters represent differences between treatments (P < 0.05). Data are presented as mean ± SEM.

The kinetics of change in the ATP content were measured to assess the effect of vitrification on mitochondrial function of embryos after 24 h of warming. While the ATP content of vitrified-warmed embryos immediately reduced after warming (P < 0.05), the level of ATP gradually increased during the 24 h incubation post-warming; however, it failed to reach the level observed in fresh embryos (Fig 1). Furthermore, we examined the mitochondrial DNA integrity by real-time PCR and found that the vitrification-induced damage of mitochondrial DNA was higher for long mitochondrial sequence than for short mitochondrial genome sequence. Relative Mt-number determined by real-time PCR targeting the short sequence was similar between fresh and vitrified-warmed embryos (Fig 2A), whereas that determined by real-time PCR targeting the long sequence was significantly lower for vitrified-warmed embryos (Fig 2A, vitrified embryo: 0.39 ± 0.05, fresh embryo; 1.0 ± 0.22, P < 0.05). In addition, the ratio of the Mt-number (long sequence/short sequence) for vitrified-warmed embryos was significantly lower than that for fresh embryos (Fig 2B, vitrified embryo: 0.37 ± 0.05, fresh embryo: 1.0 ± 0.22, P < 0.05).

### Vitrification reduced the developmental ability of mitochondria, and quantity of post-warming embryos

**Fig 3:**
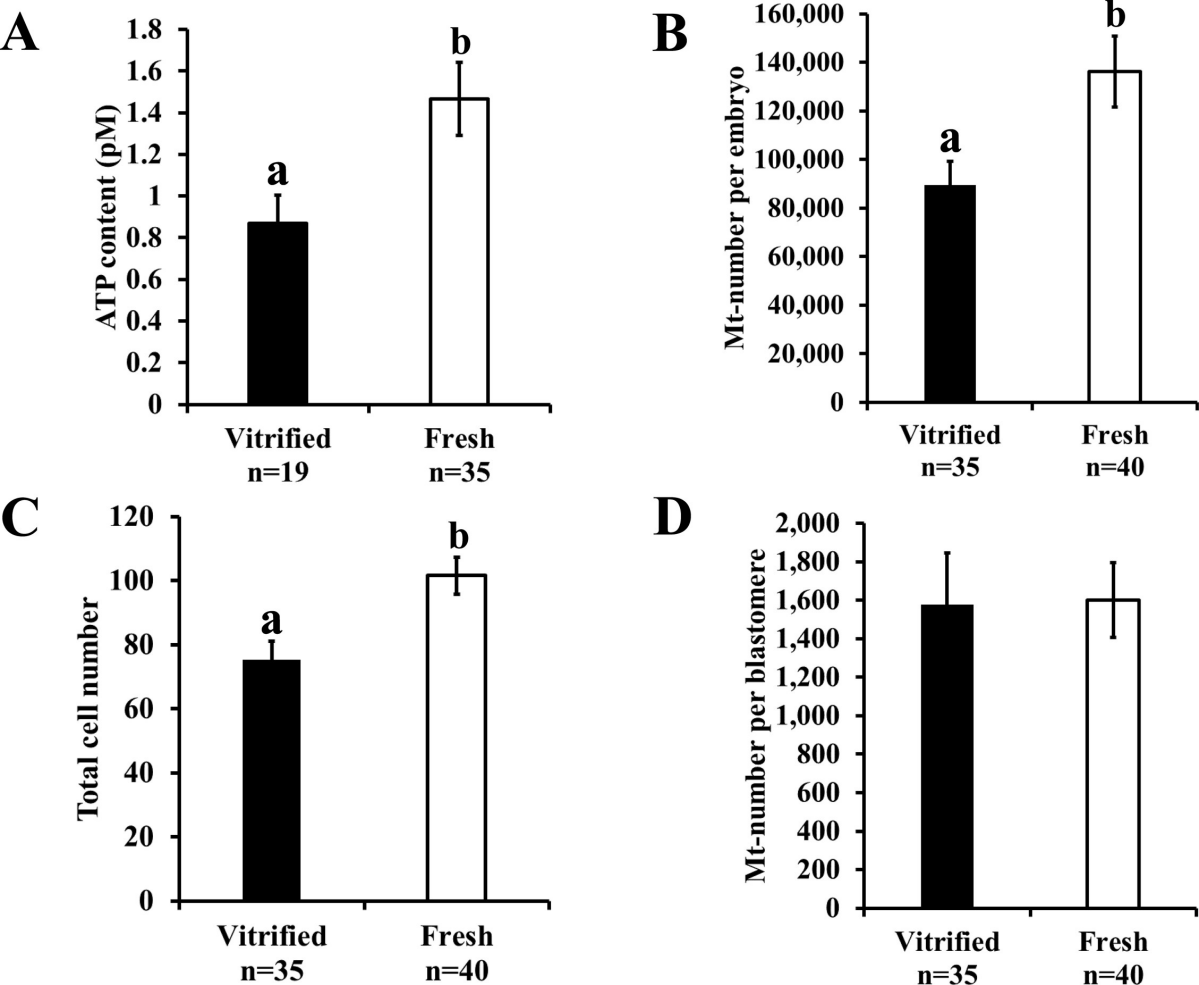
Effect of vitrification on mitochondrial number and function in blastocysts. A) ATP content, B) Mt-number per embryo, C) total cell number, and D) Mt-number per blastomere. Fresh and vitrified-warmed embryos were cultured for 5 days, and the randomly selected blastocysts were assessed. Bars with different letters represent differences between treatments (P < 0.05). Data are presented as mean ± SEM.

Vitrification decreased the blastulation rate (Table 1, P < 0.05) in post-warming embryos. The ATP content was significantly lower in the blastocysts derived from the vitrified-warmed embryos than those derived from the fresh embryos (Fig 3A, vitrified embryo: 0.87 ± 0.14 pM, fresh embryo: 1.47 ± 0.18 pM, P < 0.05). Moreover, the Mt-number per embryo reduced following vitrification (Fig 3B, vitrified embryo: 89,572.7 ± 9,631.8, fresh embryo: 136,174.4 ± 14,560.3, P < 0.05). We determined the total cell number and Mt-number in the same blastocyst (measurements of the Mt-number in embryos and blastomeres) and found that the total cell number in the blastocysts derived from the vitrified-warmed embryos was low (Fig 3C, vitrified embryo: 75.3 ± 5.7, fresh embryo: 101.6 ± 5.8, P < 0.05); however, the Mt-number per blastomere was similar between the vitrified-warmed and fresh embryos (Fig 3D, vitrified embryo: 1,576 ± 268.2, fresh embryo: 1,600 ± 194.2).

**Table 1:** Effect of vitrification on the embryonic developmental rate till the blastocyst stage.

Group	Embryo	Trial	Total (%, mean ± SE)
**Vitrification**	**320**	**32**	**96 (30.0 ± 3.0)**^**a**^
**Fresh**	**270**	**27**	**118 (43.7 ± 2.6)**^**b**^

^a,b^Means with different superscripts differ from each other (P < 0.05).

### Resveratrol improved embryonic development by maintaining the ATP content and reducing the Mt-number of blastocysts and blastomeres

**Fig 4:**
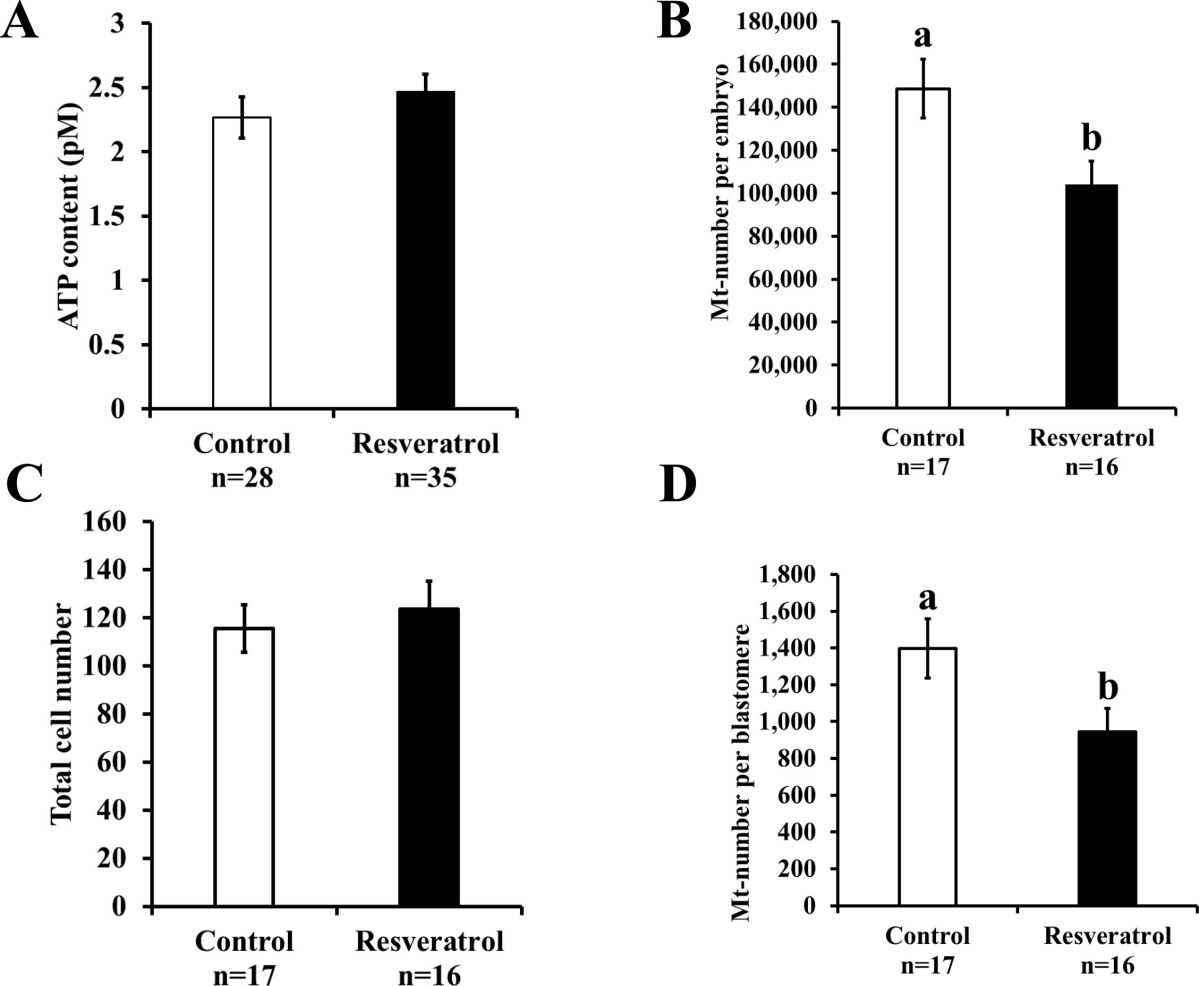
Effect of resveratrol on the characteristics of blastocysts derived from vitrified-warmed embryos. Vitrified-warmed embryos were divided into two groups and incubated in a medium containing either vehicle (control) or resveratrol (0.5 μM) for 5 days. A) ATP content, B) Mt-number per embryo, C) total cell number, D) Mt-number per blastomere. Bars with different letters represent difference between treatments (P < 0.05). Data are presented as mean ± SEM.

**Fig 5:**
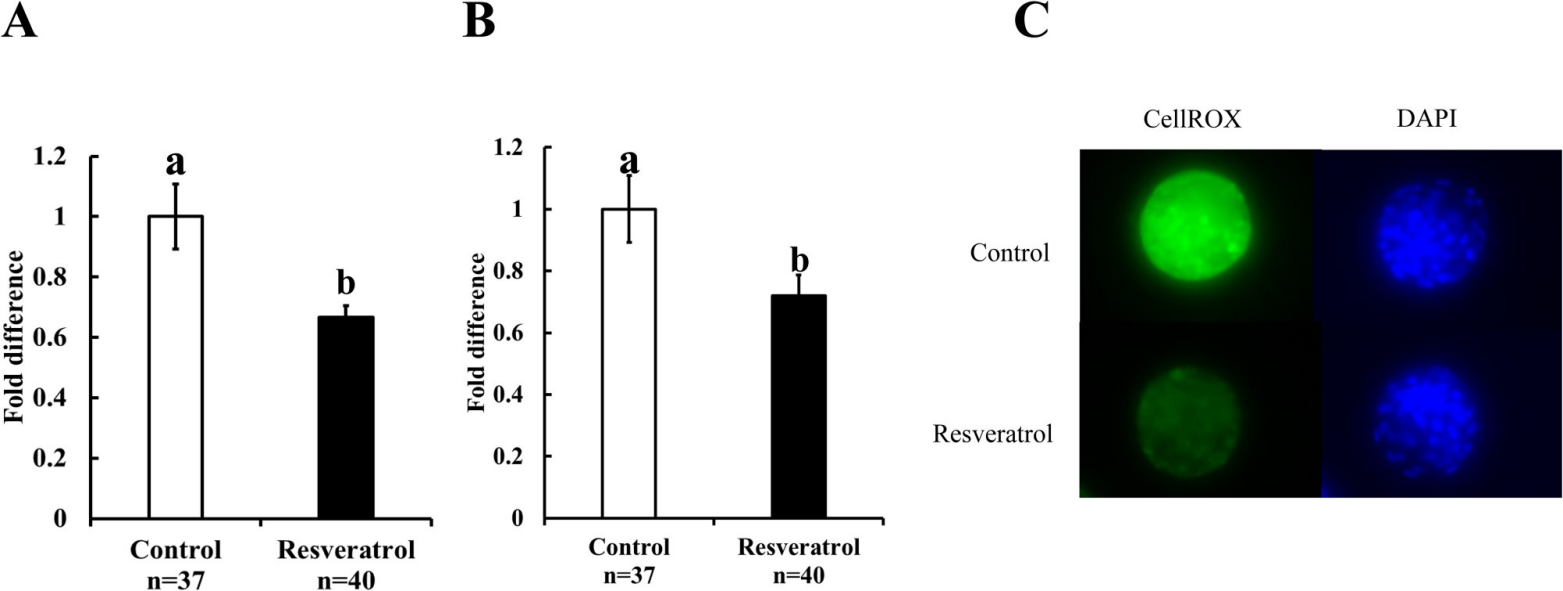
Effect of resveratrol on the ROS content in blastocysts and blastomeres. Vitrified-warmed embryos were cultured with either vehicle (control) or resveratrol (0.5μM), and the blastocysts were stained with CellROX. A) ROS content per blastocyst, B) ROS content per blastomere, C) Representative images of CellROX-stained blastocysts. The average fluorescence intensity of vitrified-warmed embryos cultured with vehicle (control) was defined as 1.0. Bars with different letters represent difference between treatments (P < 0.05). Data are presented as mean ± SEM.

Supplementation of the culture medium with resveratrol (0.5 μM) improved the blastulation rate (Table 2, P < 0.05); however, the ATP content remained similar in both the groups (Fig 4A; control: 2.2 ± 0.2 pM and resveratrol: 2.4 ± 0.1 pM). Although resveratrol decreased the Mt-number per blastocyst, the total cell number of blastocysts remained similar in both the groups (Fig 4B, Mt-number per embryo: control, 148,642.0 ± 13,720.7 and resveratrol, 104,022.9 ± 10,994.2; P < 0.05; Fig 4C; total cell number: control, 115.5 ± 9.9 and resveratrol, 123.5 ± 11.7). We found significantly low Mt-number per blastomere in the embryos cultured in the resveratrol-supplemented medium (Fig 4D; control: 1,397.4 ± 161.6 and resveratrol: 944.0 ± 127.3; P < 0.05). Furthermore, we also examined the mitochondrial quality by determining the ROS content of embryos. The ROS content in blastocysts (Fig 5A; control: 1.0 ± 0.11 and resveratrol: 0.7 ± 0.04; P < 0.05) and blastomeres (Fig 5B; control: 1.0 ± 0.11 and resveratrol: 0.7 ± 0.07; P < 0.05) was low for embryos cultured in the presence of resveratrol.

**Table 2:** Effect of resveratrol on the developmental ability of vitrified-warmed embryos during early developmental stage.

Group	Resveratrol (μM)	No. of embryo	No. of trial	Total (%, mean ± SE)
**Control**	**0**	**259**	**26**	**88 (34.0 ± 2.6)**^**a**^
**Resveratrol**	**0.5**	**259**	**26**	**115 (44.4% ± 3.2)**^**b**^

^a,b^Means with different superscripts differ from each other (P < 0.05).

### Resveratrol increased the cell-free mitochondrial DNA content in spent culture medium

**Fig 6:**
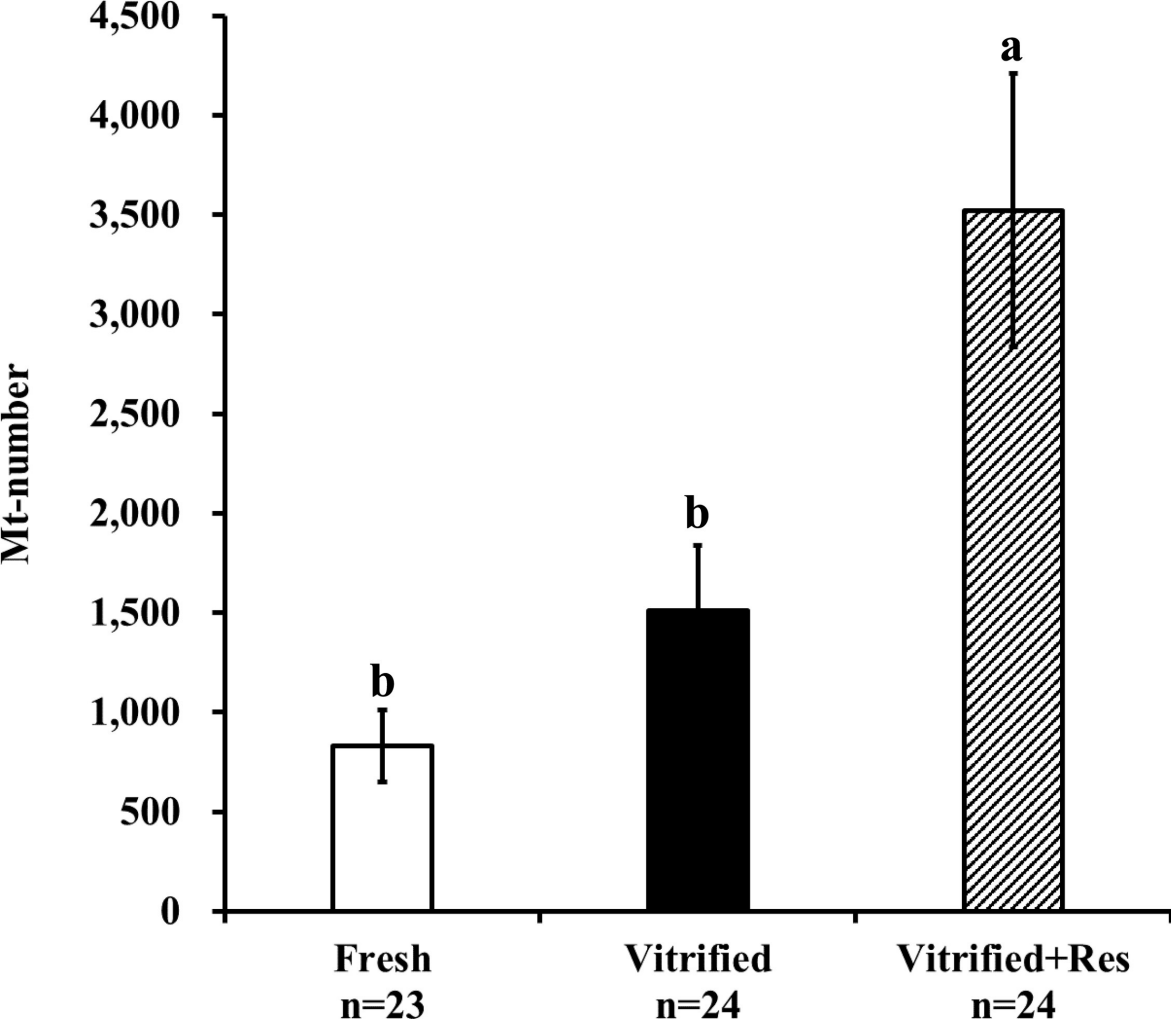
Effect of resveratrol on the cell-free mitochondrial DNA copy number in the spent culture medium of blastocysts. The spent culture medium of the blastocysts derived from fresh or vitrified-warmed embryos cultured with or without resveratrol (0.5 μM) for 5 days was examined. Bars with different letters represent differences between treatments (P < 0.05). Data are presented as mean ± SEM.

Fresh embryos were individually cultured in a medium supplemented with the vehicle, and post-warmed embryos were individually cultured with either resveratrol or the vehicle. After 5 days of incubation (day 7 post-IVF), the culture medium carrying the developed blastocysts was collected. A total of 24 spent culture medium samples were used to determine the amount of cell-free mitochondrial DNA in each experimental group. The medium alone (not used for embryo culture) contained very low levels of cell-free mitochondrial DNA (9.9 ± 2.4 per 5 μL culture droplet, n = 24), derived probably from the FCS supplementation to the IVC medium. As shown in Fig 6, the cell-free mitochondrial DNA content of the spent culture medium for fresh embryos (without vitrification) was 830.7 ± 181.5 per 5 μL of culture droplet which increased to 1,511.4 ± 326.0 per 5 μL of culture droplet for the blastocysts derived from the vitrified-warmed embryos. However, no significant difference was observed between the two groups (P > 0.05). The spent culture medium carrying vitrified-warmed embryos developed in the presence of resveratrol contained significantly higher level of cell-free mitochondrial DNA (3,522.8 ± 686.7 per 5 μL of culture droplet, P < 0.05).

### Resveratrol decreased the levels of dsDNA and mitochondrial membrane proteins (TOMM20) in post-warming embryos

**Fig 7:**
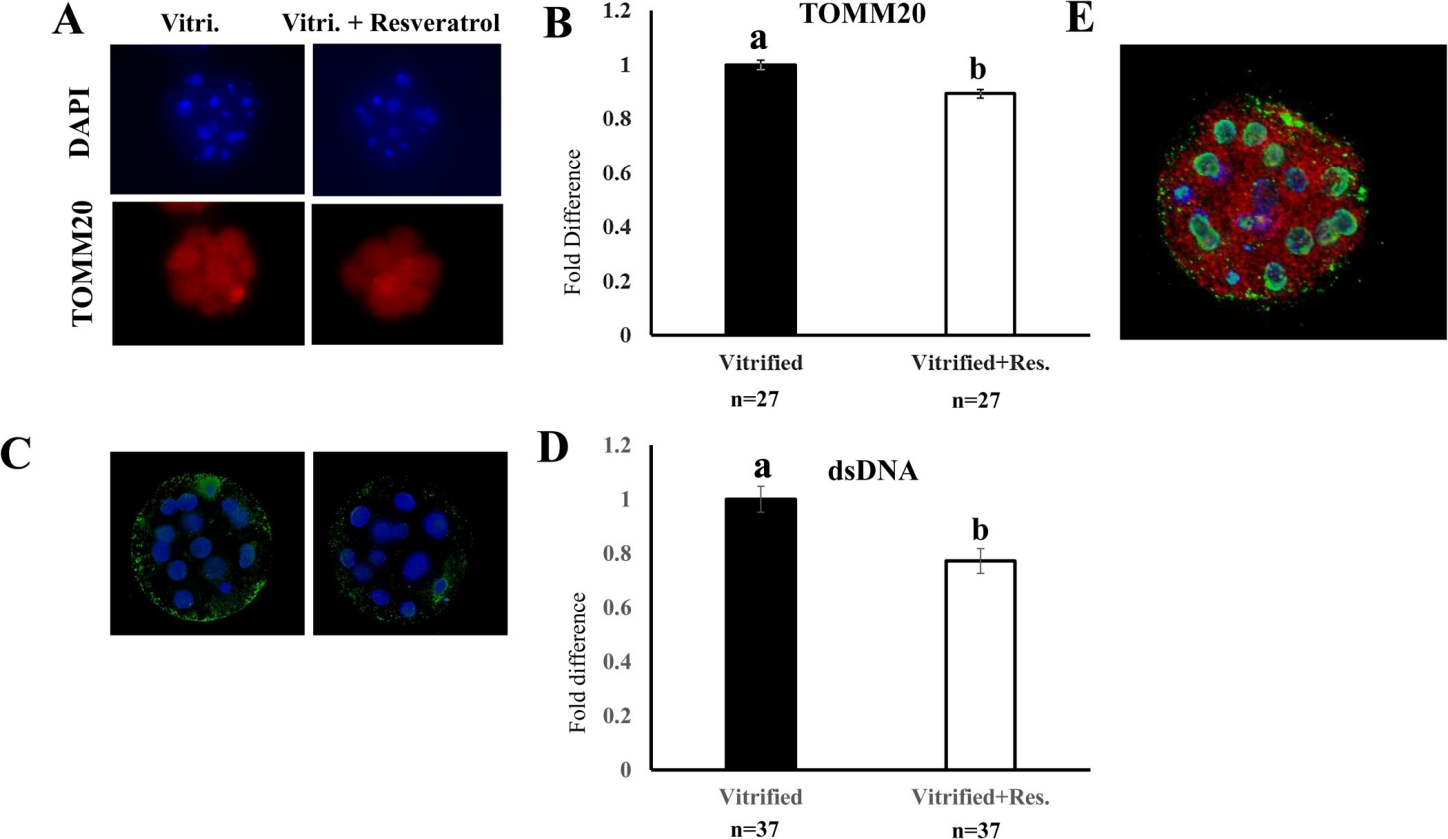
Effect of resveratrol supplementation of the culture medium on TOMM20 and double-stranded DNA (dsDNA) levels in vitrified-warmed embryos. Vitrified-warmed embryos were cultured with or without (0 or 0.5 μM) resveratrol for 24 h, and the embryos were stained for TOMM20 (an outer mitochondrial membrane protein) and dsDNA. A) and C): Representative images of embryos stained with antibodies against TOMM20 and dsDNA, respectively, B) and D): Levels of TOMM20 (n = 27) and dsDNA (n = 37), respectively. Levels of TOMM20 and dsDNA in vitrified-warmed embryos cultured without resveratrol (vehicle) were defined as 1.0. E) A representative image of embryos stained for TOMM20 (red), dsDNA (green), and nucleus (blue); ten stacked images of 0.03 μm thickness captured in the equatorial region of the embryo. Bars with different letters represent differences between treatments (P < 0.05). Data are presented as mean ± SEM.

Twenty-four hours after warming, the supplementation of the culture medium with resveratrol decreased the levels of both TOMM20 (Fig 7A and 7B) and dsDNA (Fig 7C and 7D) in vitrified-warmed embryos. Fig 7E shows a representative embryo stained with DAPI (nucleus, blue) and antibodies against dsDNA (green) and TOMM20 (red). Although the extranuclear dsDNA (green) was found distributed throughout the embryo, the highest levels were observed in the peripheral regions. In contrast, TOMM20 was evenly distributed throughout each embryo.

## Discussion

The present study demonstrates that the vitrification at the early developmental stage of embryos results in the decrease in the mitochondrial functions and mitochondrial DNA integrity. The consequences include the production of blastocysts with a low total number of blastomeres and mitochondria. In addition, we showed that resveratrol improved the development of post-warming embryos possibly through mitochondrial clearance, which corresponded to a decrease in Mt-number, mitochondrial protein, and dsDNA of the embryos and an increase in cell-free mitochondrial DNA in the spent culture medium of the resultant blastocysts.

Vitrification of oocytes and pre-implantation embryos has been reported to decrease their developmental ability [26–28] and reduces the ATP content and total cell number of the resultant blastocysts [15, 28]. Zhao XM et al. [29] monitored the ATP content in post-warming oocytes for 8 h and reported it to be consistently low for vitrified-warmed oocytes. In line with these reports, we found that post-warming embryos had low ATP content and low developmental rate up to the blastocyst stage, with a low total cell count of blastocysts. Furthermore, the resultant blastocyst showed low Mt-number, although the Mt-number per blastomere was similar. Based on these results, we suggested that one of the reasons for the low ATP content of blastocysts may be the low number of blastomeres per blastocyst. A previous study had reported that the mitochondrial number in human embryos does not change till the morula stage but may increases in the blastocyst stage embryos [30]. In addition, the Mt-number decreases during the early embryonic development and increases in the blastocyst stage [31]. These reports suggest that vitrification could induce mitochondrial dysfunction; however, the activity of mitochondrial degeneration and biosynthesis would be low during early embryonic development, and the number of mitochondria per blastomere would be similar between fresh and vitrified-warmed embryos. Vitrification has been shown to cause changes in the mitochondrial morphology and functions in sheep [32]. In the present study, vitrification-induced mitochondrial damage was assessed by real-time PCR using two primer sets targeting short and long mitochondrial sequences. In a previous study, DNA integrity of human neuroblastoma cells was evaluated using real-time PCR targeting long and short mitochondrial sequences [33]. We found low Mt-number by real-time PCR targeting the long mitochondrial sequence, indicating that the frequency of mitochondrial DNA damage was high in vitrified-warmed embryos. This study is the first to report the reduction in the mitochondrial DNA integrity following vitrification of embryos. However, the fate of the damaged mitochondria remains unclear.

**Fig 8:**
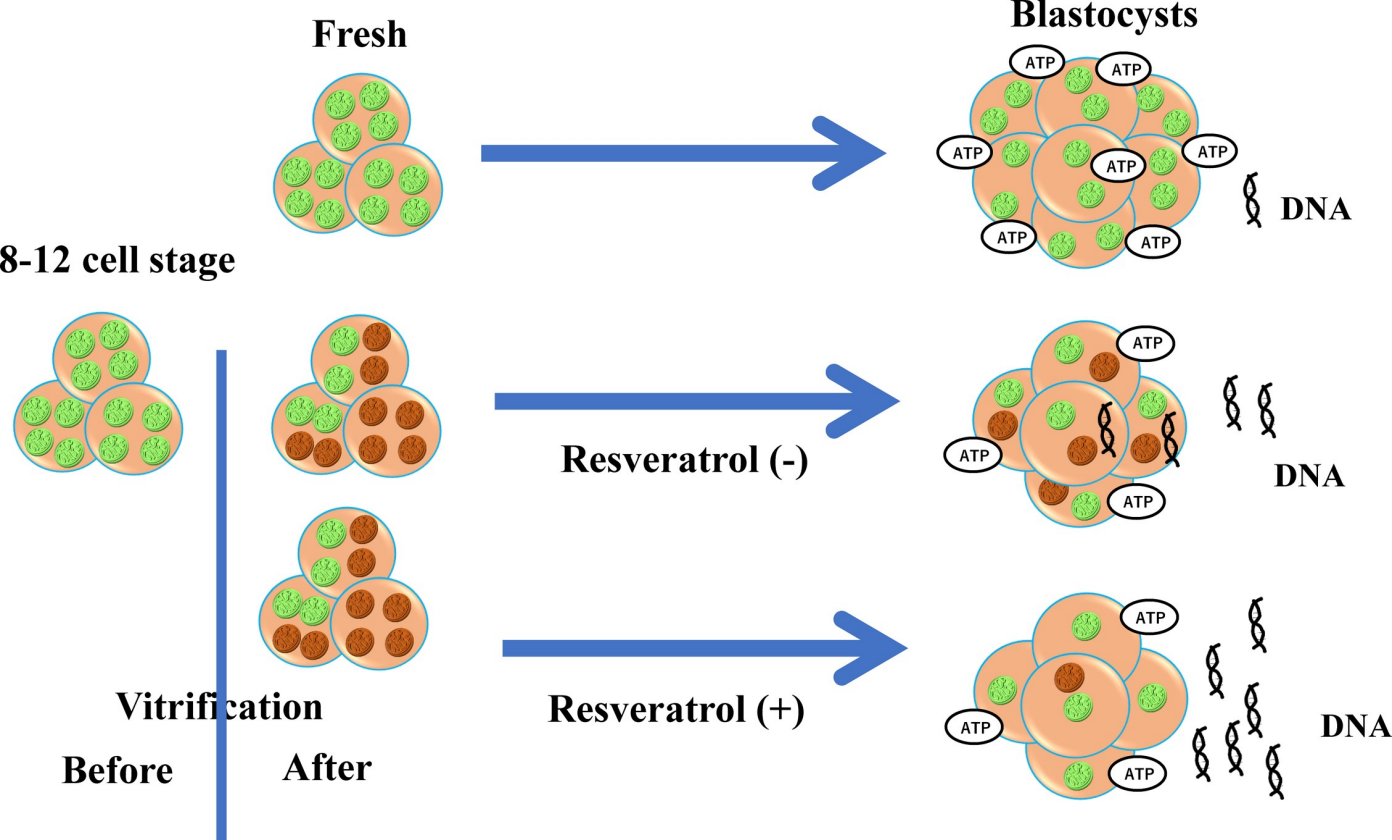
Schematic diagram of the effect of resveratrol on mitochondria in blastocysts derived from post-warming embryos. Before vitrification, the blastomeres of embryos (left side) contained healthy mitochondria (green dots), but vitrification induced mitochondrial damage (brown dots). The blastocysts derived from the vitrified-warmed embryos had low numbers of blastomeres; however, these blastocysts still contained similar numbers of mitochondria per blastomere as compared to the blastocysts derived from fresh embryos (non-vitrified, upper right). Supplementation of the culture medium with resveratrol improved the process of embryonic development up to the blastocyst stage, and the resultant blastocysts had similar numbers of blastomeres but lower numbers of mitochondria per blastomere (lower right). This reduced mitochondrial number corresponded to an increase in cell-free mitochondrial DNA in the spent culture medium and a decrease in dsDNA in the embryo.

The decomposition of damaged mitochondria is essential for maintaining cellular homeostasis [34, 35]. Therefore, in post-warming embryos, the damaged mitochondria may be eliminated during the subsequent process of embryonic development. Resveratrol is a potent activator of SIRT1 and can enhances the mitochondrial quality required for the regulation and maintenance of cellular homeostasis [6, 36]. In porcine oocytes, resveratrol treatment could induce mitochondrial degeneration and biosynthesis, thereby improving the developmental rate [2]. In the present study, the supplementation of the embryo culture medium with resveratrol enhanced the developmental rate till the blastocyst stage. Previous reports have highlighted the beneficial effects of resveratrol on the embryonic development of porcine and bovine embryos had been reported previously [22, 37]. In the present study, the incubation of 8-cell stage bovine embryos in the resveratrol-containing medium resulted in an increase in the expression level of SIRT1 in the resultant blastocysts [22]. Furthermore, resveratrol had no effect on the mitochondrial number and total cell number of blastocysts. In the present study, resveratrol was found to decrease the ROS content and Mt-number of blastocysts and blastomeres without reducing the ATP content. These results suggest that resveratrol induced the clearance of damaged mitochondria from the embryos. Taken together, resveratrol could reduce Mt-number by promoting the active removal of the damaged mitochondria from the vitrified-warmed embryos, thereby serving as a potent activator of SIRT1 that stimulates autophagy and mitochondrial biogenesis [38, 39]. However, it is very difficult to assess the kinetics of damaged mitochondria in the embryos of large animals. In the present study, we also determined the amount of cell-free mitochondrial DNA content in the spent culture medium and found that resveratrol increases the content of cell-free mitochondrial DNA in the spent culture medium of blastocysts derived from vitrified-warmed embryos. This study is the first report that demonstrates the association between cell-free mitochondrial DNA and mitochondrial degeneration in embryos. Resveratrol-induced decrease in the Mt-number of embryos and increase in the cell-free mitochondrial DNA content in the spent culture medium were in agreement with the decrease in TOMM20 and dsDNA levels in resveratrol-treated vitrified-warmed embryos. The schematic diagram in Fig 8 represents our proposed hypothesis based on these results. Following cryopreservation-induced mitochondrial damage, blastomeres with cellular damage greater than a certain threshold would degenerate, whereas the remaining blastomeres with relatively low mitochondrial damage would develop till the blastocyst stage, despite having damaged-mitochondria. However, mitochondrial degeneration may be enhanced following incubation of the embryos in a medium containing resveratrol, which may reduce the mitochondrial number and ROS content but maintains the ATP level in embryos. Furthermore, the mitochondrial degeneration would correspond to an increase in cell-free mitochondrial DNA content in the spent culture medium. A recent study with bovine blastocysts has reported that the enhanced mitochondrial generation and degeneration could support the recovery of the cryopreserved and thawed embryos [23]; the pretreatment of blastocysts with resveratrol was found to enhance SIRT1 expression and improved mitochondrial quantity, developmental ability, and pregnancy rate following embryo transfer.

In conclusion, vitrification may negatively affect mitochondrial functions and DNA integrity and reduced embryonic development. Resveratrol treatment may improve embryonic development post-warming, possibly through the active removal of the damaged mitochondria.

## Supporting information

S1 Dataset (XLSX)
